# Multiscale Fatigue Performance Evaluation of Hydrated Lime and Basalt Fiber Modified Asphalt Mixture

**DOI:** 10.3390/ma16103608

**Published:** 2023-05-09

**Authors:** Hang Diao, Tianqing Ling, Zhan Zhang, Bo Peng, Qiang Huang

**Affiliations:** 1 School of Civil Engineering, Chongqing Jiaotong University, Chongqing 400074, China; 2 Sichan Institute of Building Research, Chengdu 610081, China; 3 School of Materials Science and Engineering, Chongqing Jiaotong University, Chongqing 400074, China

**Keywords:** hydrated lime, basalt fiber, thermal aging, fatigue properties, self-healing performance

## Abstract

Long-life pavement construction is an important research direction for sustainable road development. Fatigue cracking of aging asphalt pavement is one of the main reasons that affects its service life, and improving the fatigue resistance of aging asphalt pavement has become a key factor in promoting the development of long-life pavement. In order to enhance the fatigue resistance of aging asphalt pavement, hydrated lime and basalt fiber were selected to prepare a modified asphalt mixture. The resistance to fatigue is evaluated by the four-point bending fatigue test and self-healing compensation test, based on the energy method, the phenomenon-based approach, and other methods. The results of each method of evaluation were also compared and analyzed. The results indicate that the incorporation of hydrated lime can improve the adhesion of the asphalt binder, while the incorporation of basalt fiber can stabilize the internal structure. When incorporated alone, basalt fiber has no noticeable effect, while hydrated lime significantly improves the fatigue performance of the mixture after thermal aging. Mixing both ingredients produced the best improvement effect under various conditions, with a fatigue life improvement of 53%. In the multi-scale evaluation of fatigue performance, it was found that the initial stiffness modulus was unsuitable as a direct evaluation index of fatigue performance. Using the fatigue damage rate or the stable value of dissipated energy change rate as an evaluation index can clearly characterize the fatigue performance of the mixture before and after aging. The self-healing rate and self-healing decay index clearly reflected the fatigue damage healing process under repeated loading and could be used as relevant indices for evaluating the new-scale fatigue performance of asphalt mixtures.

## 1. Introduction

In recent years, with the increase in traffic volume on roads, the frequent occurrence of fatigue cracking in asphalt pavements has hindered normal passenger and cargo transportation, reduced the pavement’s service life, increased road repair and maintenance costs, and severely affected both the economic benefits and the quality of traffic services [1,2,3]. The growth of vehicle load, as well as the emergence of the asphalt aging phenomenon, has resulted in the fatigue cracking of asphalt concrete pavements and reduced the service life of pavements; in particular, fatigue cracking and thermal oxygen aging are important reasons affecting the life of asphalt pavement [4,5,6]. Garbowski et al. [7,8] developed pavement damage identification tools to facilitate the timely detection and maintenance of pavements. This has become an important direction for the global development of a sustainable development strategy to improve the heat-resistant aging performance of asphalt pavement, decrease fatigue cracking, and increase the working life of roads, which is of great significance for transportation construction.

Existing research results have shown that fatigue damage of asphalt mixtures is mainly concentrated in the asphalt and asphalt mortar [9,10]. However, during mixing, paving, and use, asphalt and asphalt mortar will undergo thermal oxygen aging, severely affecting their fatigue performance. In engineering, additives are usually added to the mixture, as well as fibers, to improve their high-temperature resistance [11,12]. Among them, the incorporation of basalt fiber can suppress the occurrence and extension of fatigue cracks in asphalt pavement, improving its fatigue performance [13,14,15], with an especially significant improvement in crack resistance under high-temperature oxidation conditions [16]. The incorporation of hydrated lime, due to its good physical adsorption properties and high chemical activity, can inhibit the aging of asphalt and change the high and low-temperature properties of the asphalt mixture [17,18,19,20]. Das et al. [21] conducted experiments that show that replacing 20% of the mineral powder with hydrated lime in asphalt significantly improves its high-temperature aging resistance. Mwanza et al. [22] found that the softening point of asphalt gradually increases with the addition of hydrated lime, while the ductility and penetration decrease. Mohammad et al. [23] conducted a rheological performance analysis and concluded that the addition of hydrated lime enhances the permanent deformation properties and fatigue durability of asphalt mastic. Davar et al. [24] demonstrated that incorporating only basalt fibers into asphalt mixtures did not significantly change its fatigue life. Kathari et al. [25] showed that incorporating basalt fibers improved the high-temperature deformation resistance of asphalt mortar. Currently, many studies evaluate the fatigue properties of asphalt mixtures using a single theory, which makes comparisons difficult due to a lack of control over the results. In order to address this issue, numerous domestic and foreign scholars have conducted research. Amir Izadi et al. [26] accurately characterized the fatigue properties of asphalt mixtures at different aging states using the dissipated energy method, and the energy dissipated during repeated loading can be accurately characterized in terms of its fatigue behavior. Liu et al. [27] evaluated the fatigue characteristics of different aging states of asphalt concrete at various loading frequencies using the S-N fatigue equation. Lv et al. [28] found that aging had the greatest impact on the initial elastic modulus of asphalt mixtures, and there was a good correlation between fatigue life and the rate of dissipated energy change stabilization values. Sun et al. [29] proved that the self-healing performance of asphalt mixtures has a strong correlation with their fatigue resistance.

In summary, hydrated lime and basalt fibers were used as additives to prepare modified asphalt mixtures, and their fatigue properties were studied. Four-point bending fatigue tests and self-healing compensation tests were conducted, and the fatigue performance was evaluated by using phenomenological and energy-based methods at multiple scales. The applicability of different evaluation indicators was explored, and the self-healing index attenuation rate was introduced to evaluate the fatigue performance. This study aimed to provide a comprehensive evaluation of the fatigue properties of modified asphalt mixtures and to evaluate their fatigue resistance through the self-healing index attenuation rate.

## 2. Materials and Methods

### 2.1. Raw Materials

**Figure 1:**
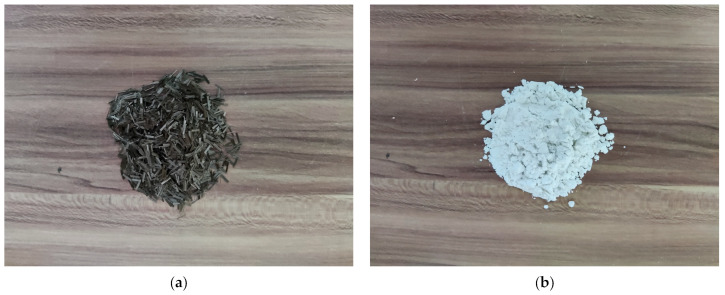
Basalt fiber and hydrated lime samples. (**a**) basalt fiber; (**b**) hydrated lime.

The asphalt used in this experiment is the base asphalt that meets the specified requirements, and its main technical specifications are shown in Table 1. Basalt fibers with a length of 6 mm were selected, and their basic performance parameters are shown in Table 2. The coarse and fine aggregates were composed of locally sourced limestone and stone chips. The filler used was hydrated lime powder and ordinary ground mineral powder of limestone. The specific parameters of the filler’s specific surface area and particle size are shown in Table 3, and the basalt fiber and hydrated lime samples are shown in Figure 1.

### 2.2. Asphalt Mixture Ratio Design

In accordance with the preliminary experiment, the optimal oil-to-rock ratio was determined to be 5.1%, and the dense grading AC-13 was used, with the composition of the grading shown in Table 4.

### 2.3. Preparation and Aging of Specimens

According to the previous experiment, we selected a 0.8 powder-to-rubber ratio; hydrated lime (HL) blending of 20% to replace the mineral powder (accounting for the mass of mineral powder); and basalt fiber (BF) blending of 0.3% (accounting for the total mass of asphalt mixture). For the preparation of a variety of asphalt mixtures, the specific types were divided as follows: not blended, blended with 20% HL, blended with 0.3% BF, 20% HL + 0.3% BF (recorded as CAM, HLAM, BFAM, and HLBFAM, respectively). The specific amount of blending and groupings are shown in Table 5.

**Figure 2:**
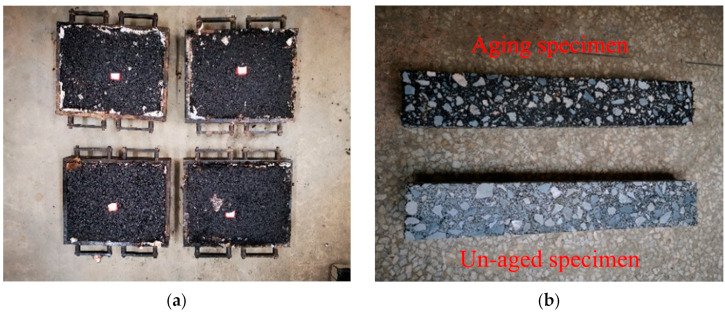
Fabrication and aging of small beam specimens: (**a**) mixture test plates; (**b**) comparison of aged and unaged specimens.

In order to more accurately simulate the aging of the asphalt mixture during the transportation, paving, and use of the actual pavement, the tests in this thesis refer to the Chinese “Test Procedure for Asphalt and Asphalt Mixture for Highway Engineering” (JTG E20-2011) [30] and the American Association of Highway Workers AASHTO-PP2 specification; the specimens to be aged were subjected to short-term aging during the mixing process of the asphalt mixture and long-term aging after using a modified mixture forming the specimen. The steps to prepare the aged specimens were as follows: the mixed asphalt mixture was placed in an oven and stirred every hour under a temperature of 135 °C. After 4 h of forced-air heating, the short-term aging was complete. Then, the aged loose materials were formed into specimens. The specimens were placed in an oven with forced-air heating at 85 °C for 120 h to complete the long-term aging. The name of the aged asphalt mixture is the name of the first four asphalt mixtures followed by the code “-A” indicating that it is the aged version. For example, CAM-A represents the aged conventional asphalt mixture. Vehicle tire rutting test specimens were prepared according to the above process, cut into small beam specimens, and used for testing, as shown in Figure 2.

### 2.4. Methodology

**Figure 3:**
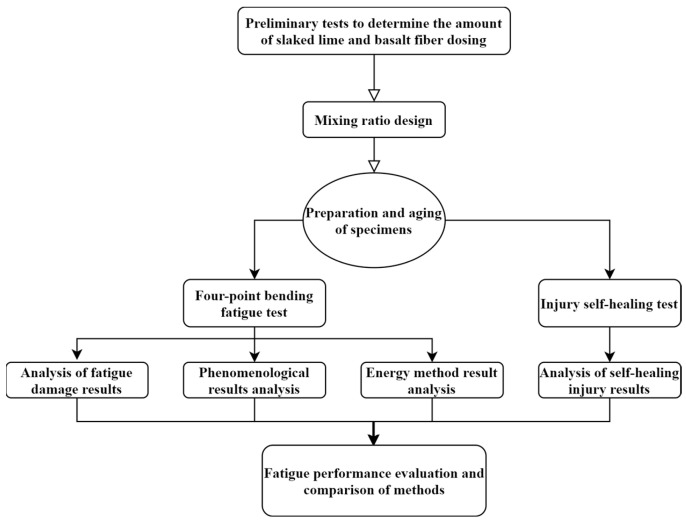
Test and analysis process.

In this study, the assessment of the fatigue performance of lime and basalt fiber modified asphalt mixes was carried out by applying various theories such as the phenomenological method and the energy method based on a four-point bending fatigue test and damage self-healing test. Figure 3 shows the test and analysis process.

#### 2.4.1. Four-Point Bending Fatigue Test

In this experiment, the fatigue performance of various asphalt mixtures prepared in the previous step was tested through four-point bending tests. In order to improve the accuracy of the experiment, a temperature close to the equivalent temperature in Chongqing was chosen, and the test temperature was 17 °C. The constant strain control mode was used with a frequency of 10 Hz and continuous sine wave loading [31,32]. The strain levels (με) of 450, 550, and 650 were determined by pre-testing the specimens. According to relevant technical specifications and research experience, the test ended when the modulus of rigidity decreased to 50% of the initial value. At the 50th loading cycle, the current modulus of rigidity was taken as the initial modulus of rigidity [24,33].

#### 2.4.2. Self-Healing Test for Injury

As the self-healing of asphalt pavements mainly takes place in the unloaded condition, a healing time was considered to be added to the two fatigue loadings, namely “fatigue–healing–fatigue” loading [34]. The asphalt mixture specimens underwent the first fatigue test to reach the corresponding damage state, and then they were placed in a specified environment for self-repair. After a certain time, a second fatigue test was performed to evaluate the self-healing performance under different conditions and use it as an indicator to assess fatigue performance.

The experiment used the four-point bending fatigue testing platform mentioned in the previous section, and the specimens were the same as before. Considering two influencing factors, temperature and initial damage level, the experiment was divided into three groups A, B, and C and conducted according to the following steps: (1) Set the test temperature at 17 °C, and all three groups use controlled strain level loading with a strain level of 650 με, a frequency of 10 Hz, and continuous sine wave loading. Groups A and B terminate the test when the modulus of rigidity decreases to 50% of the initial value, while Group C sets the termination criterion at 70%. (2) After completing the initial fatigue test, all three groups place their specimens in a constant temperature box for 5 h under different temperature conditions. Groups A and C are maintained at 17 °C, while Group B is maintained at 60 °C. After 5 h of static storage, all specimens are placed in a constant temperature box at 17 °C for 3 h until they reach the test temperature for the next step. (3) The specimens after static storage undergo another round of fatigue testing, with the relevant parameters set as in step (1). The self-healing conditions in the experiment are shown in Table 6.

## 3. Results and Discussion

### 3.1. Analysis Based on Fatigue Damage Results

**Figure 4:**
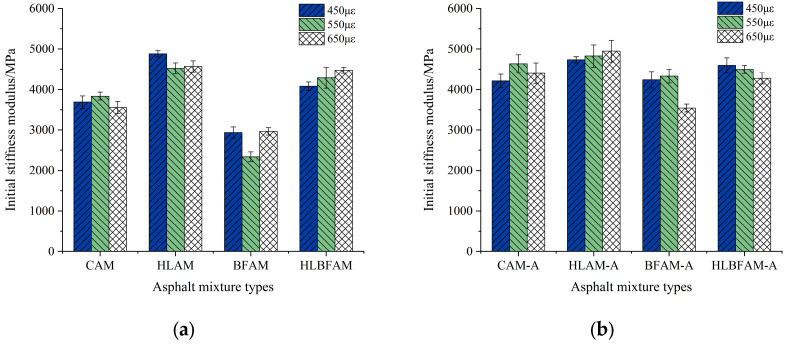
Initial stiffness modulus before and after aging of different types of asphalt mixtures: (**a**) unaged asphalt mixture; (**b**) aged asphalt mixture.

The initial modulus of rigidity is an indicator of the original bending deformation resistance of an asphalt mixture. The initial bending modulus of rigidity under three different strain levels was tested, and the results are shown in Figure 4. It can be seen that a large amount of free state asphalt becomes stable after the addition of basalt fibers, reducing the stiffness of the asphalt mixture and lowering its modulus of rigidity. Meanwhile, the rheological properties of the asphalt change with the addition of lime due to neutralizing some polar molecules, which weakens the asphalt rheological properties and increases the modulus of rigidity. After thermal aging, the polarity of asphalt internal molecules increases, and a large number of functional groups are generated inside. At the same time, the volatilization of lightweight components in the asphalt reduces their rheological properties, so the modulus of rigidity of all four mixtures increases. When hydrated lime is added, it can inhibit the high-temperature oxidation of the asphalt mixture, thus reducing the negative effects of thermal aging and lowering the modulus of rigidity. When both basalt fiber and hydrated lime are added, the modulus of rigidity increases slightly, and the amount of free-state asphalt increases as well. If fatigue performance is evaluated based solely on the modulus of rigidity, the improvement in anti-fatigue performance is not significant compared to a conventional asphalt mixture. However, it is evident from the subsequent analysis that the simultaneous incorporation of the two materials improved the fatigue resistance of the aged asphalt mixes. Therefore, it can be concluded that there is no significant correlation between the modulus of rigidity and the fatigue performance of an asphalt mixture, indicating that it is not suitable as a direct indicator for evaluating fatigue performance.

Existing research has indicated that the initial modulus of rigidity of an asphalt mixture merely represents its original bending deformation resistance, which is primarily determined by its own properties [35]. Considering that the process of fatigue failure is a long-term and complex one, it is observed that the fatigue performance and initial modulus of rigidity are not strongly correlated. Therefore, it cannot accurately reflect the fatigue damage process of asphalt mixtures. In this study, the fatigue damage rate *D* is introduced as an evaluation indicator of fatigue performance, which is expressed in Formula (1):

(1) $$D=\frac{S_{0}-S_{i}}{S_{0}}$$

where *S*_0_ is the initial stiffness modulus; *S_i_* is the stiffness modulus at the *i*-th loading.

**Figure 5:**
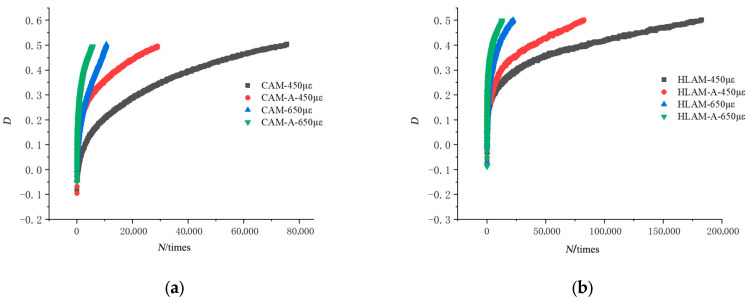

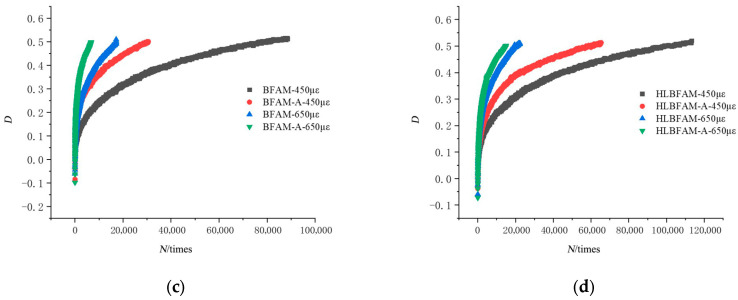
Variation trend of fatigue damage rate of various asphalt mixtures under different strain conditions: (**a**) ordinary mineral powder asphalt mixture; (**b**) hydrated lime asphalt mixture; (**c**) basalt fiber asphalt mixture; (**d**) basalt fiber + hydrated lime asphalt mixture.

The fatigue damage rate of each group of asphalt mixtures under different strains with the number of loading was plotted as shown in Figure 5.

As can be seen from Figure 5, in the early stage of loading, the fatigue damage rate of the asphalt mixture increases faster after aging. This can be explained by the hardening phenomenon that occurs within the aged asphalt mixture, which generates and develops microcracks more rapidly. However, the addition of hydrated lime effectively improves this condition. Comparing the fatigue damage curves of CAM-A and HLAM-A at the same strain level, it is found that the latter has a more gradual change trend and a longer loading cycle. This is because the addition of hydrated lime increases the viscosity of aged asphalt, enhances its adhesion to aggregates, and improves its fatigue life. In addition, the incorporation of basalt fiber into the asphalt binder can have a similar effect in asphalt mastic as reinforcement in reinforced concrete and can significantly delay the occurrence of internal fatigue cracking, as shown in Figure 5c. After thermal aging, the fatigue life of HLBFAM-A was improved by about 53% compared to CAM-A, indicating that the combined effects of both materials can delay the fatigue damage process of aged asphalt mixtures, thereby achieving a better fatigue performance, which is consistent with the conclusions made in [36].

**Figure 6:**
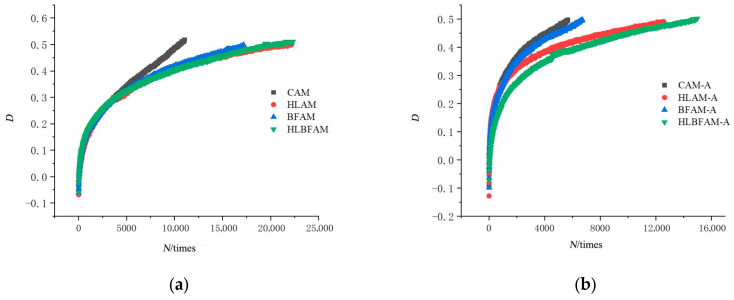
Variation trend of fatigue damage rate of various asphalt mixtures under 650 με strain conditions: (**a**) unaged asphalt mixture; (**b**) aged asphalt mixture.

For the purpose of analysis, the fatigue damage rate versus the number of loadings was separately plotted for 650 με as shown in Figure 6.

Analyzing the trend of fatigue damage rate before aging, it was found that at the same number of load cycles, the addition of basalt fibers and hydrated lime both reduced the fatigue damage rate of asphalt mixture with similar effects. Observing the change in curvature of the four curves in Figure 5b, it was found that the combined addition of hydrated lime and basalt fibers had the highest curvature, followed by the single addition of hydrated lime, the single addition of basalt fibers, and the control group. This indicates that the best anti-fatigue performance enhancement after aging was achieved with the combination of additives, and the addition of hydrated lime was more effective than basalt fibers as a single additive.

### 3.2. Analysis Based on Phenomenological Results

#### 3.2.1. Fatigue Life Analysis

**Figure 7:**
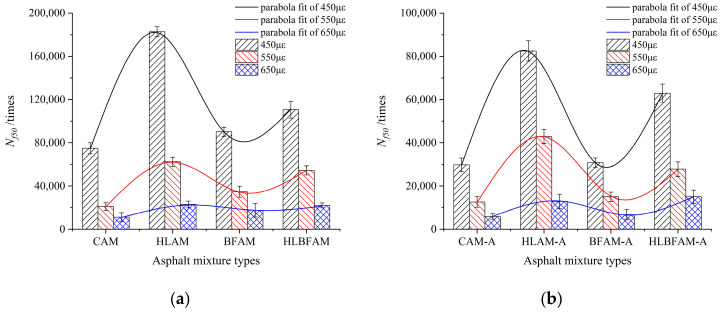
Fatigue life $N_{f50}$ before and after aging of different types of asphalt mixtures: (**a**) unaged asphalt mixture; (**b**) aged asphalt mixture.

This study conducted fatigue life tests on asphalt mixtures, and the results are shown in Figure 7. The results indicate that the addition of hydrated lime produced the most significant improvement in fatigue life, which improved by about two times compared to the common asphalt mixture under 550 με strain. The main reason for this is that hydrated lime has a large specific surface area and can adsorb the acid in the asphalt to improve its adhesion, thereby improving its fatigue characteristics. After thermal aging, the fatigue life of the asphalt mixture was reduced, and the reduction was minimal when hydrated lime was added alone, while the improvement was not significant when basalt fiber was added alone. This is because the adhesion of asphalt decreases after thermal aging, causing basalt fibers to become unable to stably embed into the asphalt, resulting in a loss of interlocking effect within the mixture. Under high strain (650 με), the mixed addition of hydrated lime and basalt fibers showed the longest fatigue life among all asphalt mixtures, thanks to the fact that hydrated lime increased asphalt adhesion while basalt fibers formed a spatial mesh structure inside the mix [37], which significantly reduced particle slip in the mastic during the crack propagation stage, and also reduced the sensitivity of the strain level in fatigue cracking.

#### 3.2.2. Analysis of Fatigue Equations

**Figure 8:**
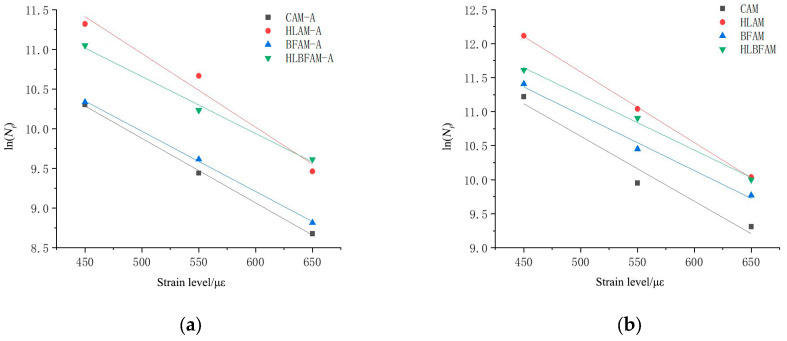
Fatigue life prediction model of asphalt mixtures under different strains: (**a**) unaged asphalt mixture; (**b**) aged asphalt mixture.

By using the results of the above experiments, the different asphalt mixes were fitted with the corresponding strain values to derive semi-logarithmic fatigue equations with a good correlation, and the fitting equations are shown in Equations (2) and (3). Figure 8 represents the model for the fatigue life prediction of asphalt mixes under different strains.

(2) $$N_{f}=c{\left(\frac{1}{\varepsilon }\right)}^{m}$$

(3) $$\ln(N_{f})=a\varepsilon +b$$

where $N_{f}$ is the fatigue life; *ε* is the strain amplitude; *a*, *b*, *c*, and *m* are constants.

The fitted semi-logarithmic fatigue equations for different kinds of asphalt mixes are shown in Table 7.

As observed in Table 7, the vast majority of asphalt mixtures have correlation coefficients close to one in each fitted result, indicating a strong correlation between fatigue life and the corresponding strain level of cyclic loading. However, different strain levels and loading frequencies produce differences in the internal stress change rate of asphalt mixtures, while the equations ignore the influence of such differences on the analysis results, which will produce variability when used as a fatigue performance assessment index.

### 3.3. Analysis of Results Based on Energy Method

#### 3.3.1. Analysis of Dissipated Energy in a Single Loading Cycle

Previous studies have shown that asphalt mixtures generate energy (dissipated energy) to resist fatigue damage during the fatigue process, which is commonly used to reflect the development of fatigue damage. In this experiment, the loading method was a sine wave, and the waveform applied to the specimen was defined as $a\sin\left(\omega t\right)$. As asphalt mixtures are viscoelastic materials, there is a phase difference φ between the generation of strain and the action of stress. Therefore, each stress action on the specimen corresponds to a strain with phase lag $b\sin\left(\omega t+\varphi \right)$. Equations (4) and (5) are defined here:

(4) $$x=a\sin\left(\omega t\right)$$

(5) $$y=b\sin\left(\omega t+\varphi \right)$$

with Equation (6):

(6) $$\cos\left(\omega t\right)=\frac{1}{\sin\left(\varphi \right)}\left(\frac{y}{b}-\frac{x}{a}\cos\left(\varphi \right)\right)$$

Combining (4), (5), and (6) yields the hysteresis equation:

(7) $${\left(\frac{x}{a}\right)}^{2}-{\left(\frac{y}{b}\right)}^{2}-\frac{2\cos\left(\varphi \right)}{ab}xy=\sin^{2}\left(\varphi \right)$$

**Figure 9:**
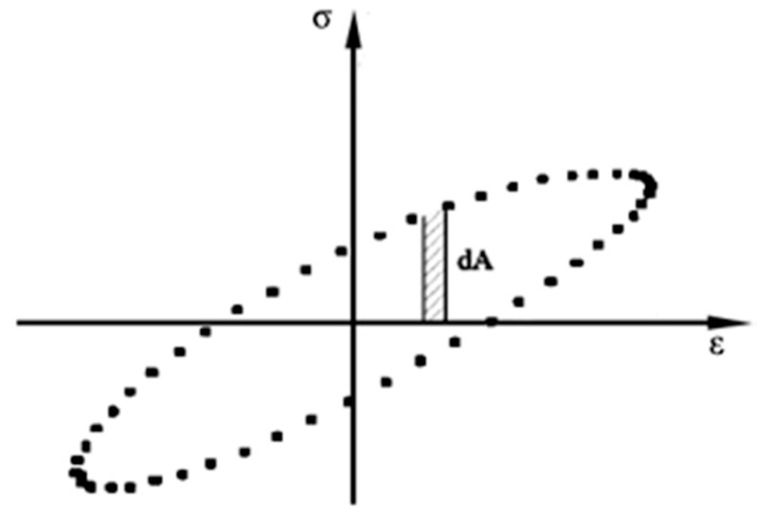
Hysteresis curve.

Figure 9 shows the hysteresis curve in one loading cycle; it can be seen that the stress and strain form a closed loop curve, and the area inside the loop curve is the dissipation energy of this loading cycle, which is calculated by Equation (8). For fatigue tests conducted using the strain control mode, the *x*-axis represents a constant strain, while the *y*-axis represents stress, which decreases continuously as the test progresses and the specimen’s fatigue damage accumulates. The circular curve will gradually shrink inward along the *y*-axis, showing that the dissipated energy of a single loading cycle is consequently reduced.

(8) $$E_{D}=\int ydx=\int_{0}^{\frac{2\pi }{\omega }}ab\omega \sin\left(\omega t+\varphi \right)\cos\left(\omega t\right)dt=\pi ab\sin\left(\varphi \right)$$

Therefore, the dissipated energy is analyzed here with a single cycle of loading, and its calculation formula is shown in Equation (9):

(9) $$E_{Di}=\pi \sigma_{ti}\varepsilon_{ti}\sin\;\varphi_{i}$$

where $E_{Di}$, $\sigma_{ti}$, $\varepsilon_{t}$, and $\varphi_{i}$ are the dissipation energy, maximum tensile stress, maximum tensile strain, and phase angle of cyclic loading up to *i* times, respectively.

**Figure 10:**
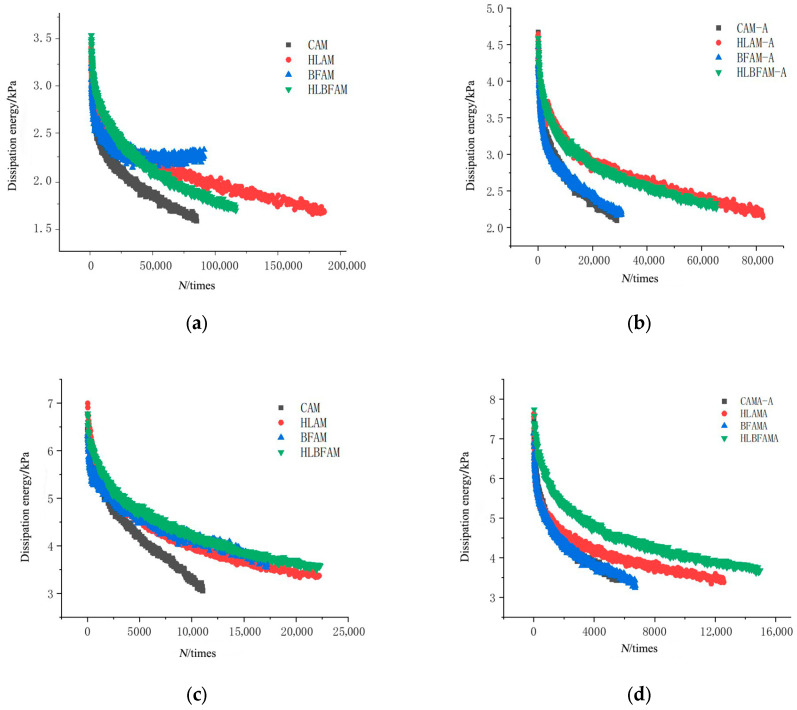
The dissipated energy varies with the number of cyclic loading: (**a**) strain 450 με, unaged; (**b**) strain 450 με, after aging; (**c**) strain 650 με, unaged; (**d**) strain 650 με, after aging.

The relationship between the dissipated energy and loading cycles for various asphalt mixtures under different strain levels is shown in Figure 10. The results indicate that the asphalt mixture fatigue loading process can be classified into two phases: in the early stages of loading, dissipated energy rapidly decreases, and microcracks gradually form within the mixture; as the number of loading cycles increases, the sensitivity of the asphalt mixture to loading decreases, and the trend of dissipated energy changes slows down towards stability. Compared with common asphalt mixtures, the addition of basalt fiber can advance the change in dissipated energy towards a stable state, indicating its internal structure is relatively stable without obvious fatigue cracking. However, after thermal aging treatment, the viscoelastic properties of the asphalt deteriorate, and the internal anchoring structure is easily damaged (causing a decrease in the adhesive strength between asphalt and basalt fiber), such that the improvement effect of the single addition of basalt fiber is not significant. Observing Figure 10b,d, the aging asphalt mixture was found to have a significantly decreased ability to resist deformation, with the initial dissipation energy increasing, and the dissipation energy decreasing rapidly along with the number of loadings, while this effect was significantly weakened by adding hydrated lime. Since the addition of hydrated lime improved the asphalt viscosity, mixing with basalt fibers—which play a significant role in the internal stabilization of the mixture—greatly improved the fatigue resistance under the effect of thermal aging; the results also indicated that the longer the change in the stability period of the dissipation energy of the asphalt mixture, the better the fatigue performance.

#### 3.3.2. Analysis of the Stable Value of the Rate of Change in Dissipated Energy

Previous studies have shown that some researchers calculate the rate of dissipated energy change for individual intervals (RDEC) evenly and use it as an evaluation index of the fatigue performance of asphalt mixtures [38]. When using RDEC to characterize the fatigue damage process of asphalt mixtures, which can be separated into a smooth phase and an accelerated damage phase, the stable value of the dissipated energy change rate (*PV*) represents the value of the dissipated energy change rate in the steady stage. The larger the *PV* value, the faster the microcracks expand inside the mixture, and the greater the fatigue damage. This paper introduces the calculation method of *PV* value proposed by Carpenter et al. [39]. The dissipated energy-loading cycle equation was fitted with a power function and the K value was calculated; the results are shown in Table 8. Then, through Equation (10), the *PV* values of different asphalt mixtures under 550 με and 650 με strain conditions were calculated, and the results are shown in Figure 10.

(10) $$PV=\frac{1-{\left(1+\frac{100}{N_{f50}}\right)}^{K}}{100}$$

where $N_{f50}$ is the fatigue life; *K* is a constant.

**Figure 11:**
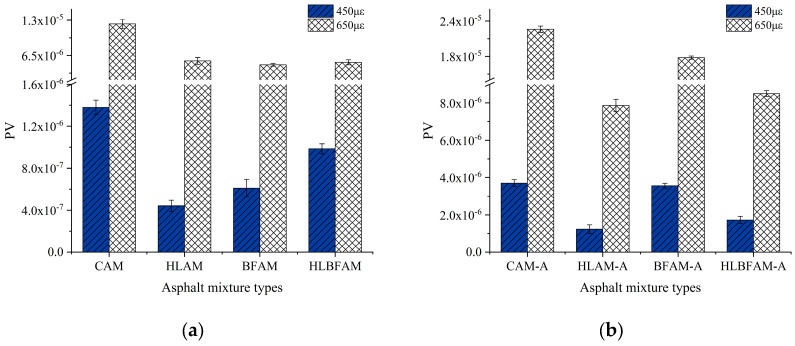
PV value of aged and unaged asphalt mixture: (**a**) unaged asphalt mixture; (**b**) aged asphalt mixture.

From Figure 11, it can be seen that under the unaged condition, the PV values of asphalt mixtures containing basalt fiber and hydrated lime are lower because their energy consumption during the loading stage is relatively small, which prolongs the time of crack formation and development to a certain extent, thereby enhancing the fatigue performance. After thermal aging, the PV value of the mixture with added hydrated lime is only 1/3 of that of ordinary asphalt mixtures, which illustrates the promotion effect of hydrated lime on the fatigue resistance improvement of aging asphalt mixtures. Observing Figure 10a, we can see that under 650 με strain conditions, the asphalt mixture with only basalt fiber has the lowest PV value, which confirms the conclusion that the addition of BF in phenomenological methods will reduce the high strain sensitivity of asphalt mixtures.

### 3.4. Analysis Based on Injury Self-Healing Results

After the self-healing test was completed, the fatigue life ($N_{f1}$), initial stiffness modulus ($S_{1}$), and stiffness modulus ($S_{e1}$) at the end of the test were recorded for the first cyclic loading, and the fatigue life ($N_{f2}$), initial stiffness modulus ($S_{2}$), and stiffness modulus ($S_{e2}$) at the end of the second cyclic loading, with the results presented in Table 9.

Since damage self-healing is a long and complex process, its effects cannot be described by a single index [40]. Therefore, the self-healing rate (*HI*) expression introduced in this study was calculated and analyzed, as shown in Equation (11):

(11) $$HI=\frac{D_{1}}{D_{2}}$$

where $D_{1}$ is the rate of decrease in the stiffness modulus at the first cyclic loading; $D_{2}$ is the rate of decrease in the stiffness modulus at the second cyclic loading.

The calculations of $D_{1}$ and $D_{2}$ in this test are shown in Equations (12) and (13):

(12) $$D_{1}=\frac{S_{1}-S_{e1}}{N_{f1}}$$

(13) $$D_{2}=\frac{S_{2}-S_{e2}}{N_{f2}}$$

**Figure 12:**
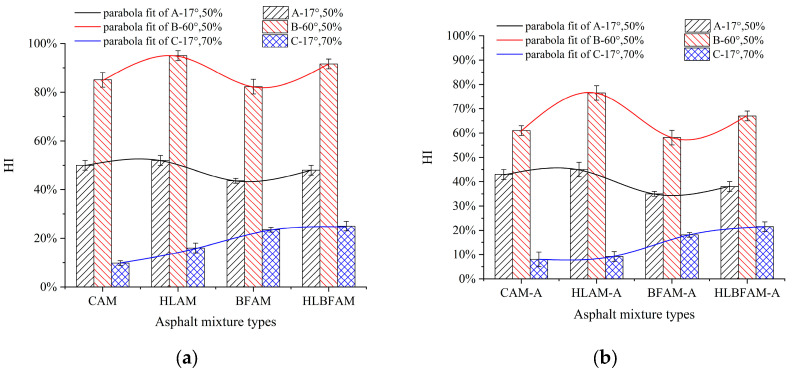
Self-healing rate of aged and unaged asphalt mixture: (**a**) unaged asphalt mixture; (**b**) aged asphalt mixture.

Figure 12 shows the self-healing rates (*HI*) of four different types of asphalt mixtures under different temperatures and damage levels. Comparing the three healing conditions, it can be found that an excessive accumulation of fatigue damage will greatly weaken the self-healing ability of asphalt mixtures. After 70% damage accumulation, the self-healing performance decreased by 75% to 90%. In addition, under the same damage conditions, the higher the temperature, the higher the self-healing rate of the asphalt mixture.

From Figure 12a, it can be seen that adding hydrated lime to the asphalt mixture has the best self-healing performance at 60 °C, with a self-healing rate of up to 96%, while there is no significant improvement at 17 °C. When the damage condition is 50%, the self-healing effect of the asphalt mixture containing only basalt fiber added is decreased. This is because the internal microcracks of the asphalt mixture are still in the stage of expansion, and the wetting and diffusion conditions between the asphalt interfaces are good at this time. The “bridging” effect of the fiber on the asphalt molecules does not have a significant promotion effect, and the uneven distribution of some fibers in the slurry will hinder their free movement and aggregation, thereby reducing their bonding performance with the aggregate. However, when the damage condition is 70%, the microcracks inside the mixture begin to expand, and the “bridging” effect of the fiber on the asphalt molecules begins to appear, producing better diffusion conditions and a better self-healing performance, with a doubled self-healing rate. Compared with the common mineral powder asphalt mixture, the asphalt mixture which incorporated both basalt fiber and hydrated lime had a better self-healing ability, and its self-healing rate increased by 6% to 15% under the larger initial damage and high temperature; in addition, the high temperature aging had no significant effect on its performance, and its self-healing property decreased the least compared with the unaged specimens in the above two cases, which had values of about 16% to 26%.

**Figure 13:**
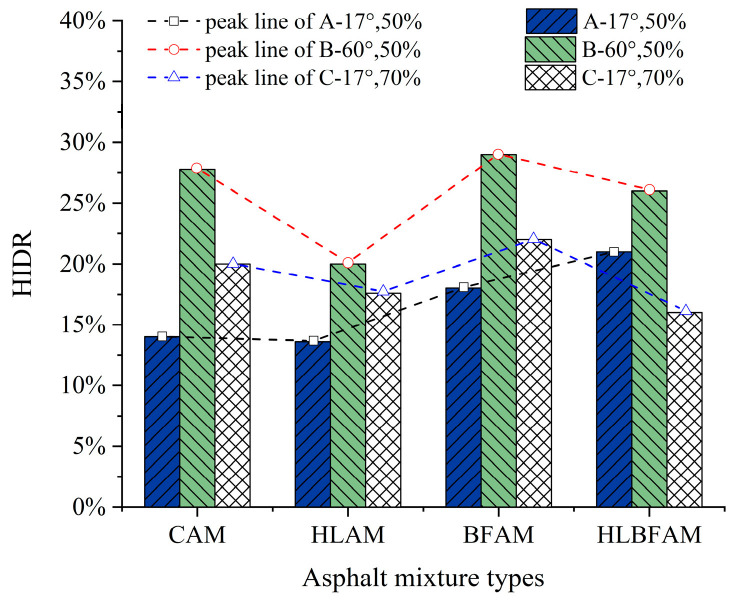
HI decay rate of different asphalt mixtures.

With the aging of the asphalt mixture, its self-healing performance has a certain degree of decline, justifying the introduction of a self-healing index decay rate (*HIDR*); the size of the *HIDR* can characterize the degree of self-healing performance decay after aging and reflect the effect of aging on the fatigue damage process. Thus, the fatigue performance analysis may use the *HIDR*, the definition of which is shown in Equation (14). The *HIDR* values of each asphalt mixture under the three healing conditions were calculated, and the results are shown in Figure 13.

(14) $$HIDR=\frac{HI-HIA}{HI}$$

where HIDR is the self-healing index decay rate; HI is the self-healing rate; HIA is the self-healing rate after aging.

The results indicate that aging has a significant impact on the self-healing ability of asphalt mixtures under high temperature and high damage conditions. The main reason is that the light components in the asphalt decrease after aging, resulting in reduced fluidity, which severely affects the self-repair ability at high temperatures. Additionally, excessive accumulation of fatigue damage can cause the internal cracks of brittle aged asphalt materials to expand, making it difficult for asphalt molecules to flow back and diffuse for repair. Meanwhile, *HIDR* also confirms that adding basalt fiber and hydrated lime to asphalt mixtures during the thermal aging process can improve the fatigue self-healing performance of asphalt mixtures, which is consistent with the conclusion obtained previously.

## 4. Conclusions

This paper conducted four-point bending fatigue tests and damage self-healing tests to measure and record various parameters of four types of asphalt mixtures during the fatigue process before and after thermal aging. Based on changes in the modulus of rigidity, phenomenological methods, energy methods, and other approaches, the fatigue behaviors of the four asphalt mixtures before and after aging were analyzed, and the following conclusions were drawn:

1. For the unaged asphalt mixture, the adhesion strength between the asphalt binder is obviously improved by the addition of hydrated lime, and its fatigue characteristics are significantly improved under high temperature, and the fatigue life is increased significantly; the addition of basalt fiber plays an anchoring role in the mixture, which can enhance its sensitivity to changes in load and strain levels and make the internal structure more stable; the fatigue performance is improved well when the two are mixed.
2. For the asphalt mixture after thermal aging, under low strain conditions, single lime has a better fatigue resistance and its fatigue life is about three times that of the ordinary mix, but no significant effect was observed when blended with basalt fiber alone; here, we believe that the decline in fatigue performance was a result of asphalt aging due to reduced mobility, being more easily wrapped in the fiber surface, asphalt wetting, and a significantly reduced diffusion capacity. When mixed with hydrated lime, basalt fiber produced a good fatigue resistance under high strain conditions, its fatigue life and self-healing performance were about two times the ordinary asphalt mixture, and the overall enhancement produced the best effect.
3. The initial stiffness modulus reflects the original flexural capacity of the asphalt mixture which only represents the initial condition of the asphalt mixture; however, fatigue damage is a long-term procedure, making the initial stiffness modulus inappropriate for evaluating the fatigue properties of asphalt mixtures subjected to long-term loading. When analyzing the asphalt mixture based on the fatigue equation, the difference in the rate of change in the internal stress of the asphalt mixture under different strain levels and loading frequencies is not taken into account, which can lead to deviations in the analysis results.
4. The results of the fatigue damage rate and the steady value of the dissipation energy change rate analysis show the similarity, and both of these can reflect the fatigue resistance characteristics well. Under complex damage conditions, the fatigue performance of the asphalt mixture is evaluated by the self-healing rate and the decay rate of the self-healing index; both indicators can fully characterize the damage healing process under repeated loading, and have a good correlation with the fatigue characteristics, which can be used as fatigue performance evaluation indicators.

## Figures and Tables

**Table 1:** Basic performance of asphalt.

Project		Detection Value	Standard Requirements	Experiment Methods
Penetration (25 °C, 100 g, 5 s)/(0.1 mm)		67	60~80	T0604
Ductility (15 °C, 5 cm/min)/(cm)		141	≥100	T0605
Ductility (10 °C, 5 cm/min)/(cm)		27	≥20	T0605
Softening point (°C)		48.5	≥45	T0606
Density (15 °C)/(g/cm^3^)		1.01	Measured value	T0603
Ductility (10 °C, 5 cm/min)/(cm)		27	≥20	T0605
Wax content (distillation method) (%)		1.8	≤2.2	T0615
After RTFOT:	Mass loss (%)	0.13	≤±0.8	T0609
	Penetration ratio (25 °C) (%)	71	≥61	T0604
	Ductility (10 °C)/(cm)	8	≥6	T0605

**Table 2:** Characteristics of basalt fiber.

Project	Index
Density/(g/cm^3^)	2.65
Fiber diameter/μm	17
Average length/mm	6 ± 0.5
Tensile strength/MPa	1050
Elastic modulus/GPa	7.6
Fracture elongation/%	3
Operating temperature/°C	−260–650

**Table 3:** Particle size and specific surface area of mineral powder and hydrated lime powder.

Type	D90 (μm)	SSA (m^2^/g)
limestone ore powder	73.8	1.13
Hydrated lime	43.2	2.48

**Table 4:** Aggregate gradation of AC-13.

**Sieve size/mm**	16.0	13.2	9.5	4.75	2.36	1.18	0.6	0.3	0.15	0.075
**Passing/%**	100	98	80	58	40	27.9	19.9	14.4	10.4	5.6

**Table 5:** Group of mixture specimens.

Specimen Types	Powder to Glue Ratio	Thermal Aging	Hydrated Lime (%)	Limestone Powder (%)	Basalt Fiber (%)
CAM	0.8	No	0	100	0
HLAM	0.8	No	20	80	0
BFAM	0.8	No	0	100	0.3
HLBFAM	0.8	No	20	80	0.3
CAM-A	0.8	Yes	0	100	0
HLAM-A	0.8	Yes	20	80	0
BFAM-A	0.8	Yes	0	100	0.3
HLBFAM-A	0.8	Yes	20	80	0.3

**Table 6:** Self-healing condition.

Group	Injury Degree (%)	Healing Temperature, Healing Time (°C, h)
A	50	17 °C, 6 h
B	50	(60 °C, 3 h) + (17 °C, 3 h)
C	70	17 °C, 6 h

**Table 7:** Different types of half logarithm fatigue equation of asphalt mixtures.

Type of Asphalt Mixture	Fatigue Equation	R^2^
CAM	$\ln\left(N_{f}\right)=-0.00956\varepsilon +15.418$	0.92991
HLAM	$\ln\left(N_{f}\right)=-0.01039\varepsilon +16.779$	0.99926
BFAM	$\ln\left(N_{f}\right)=-0.00819\varepsilon +15.049$	0.92748
HLBFAM	$\ln\left(N_{f}\right)=-0.00807\varepsilon +15.278$	0.97301
CAM-A	$\ln\left(N_{f}\right)=-0.00813\varepsilon +13.942$	0.99967
HLAM-A	$\ln\left(N_{f}\right)=-0.00928\varepsilon +15.590$	0.94338
BFAM-A	$\ln\left(N_{f}\right)=-0.00759\varepsilon +13.764$	0.99824
HLBFAM-A	$\ln\left(N_{f}\right)=-0.00738\varepsilon +14.245$	0.98707

**Table 8:** Dissipated energy fitting equation.

Strain Level (με)	Type of Asphalt Mixture	Fatigue Equation	K
450 με	CAM	$y=5.81x^{-0.103}$	−0.103
	HLAM	$y=5.29x^{-0.082}$	−0.082
	BFAM	$y=4.06x^{-0.055}$	−0.055
	HLBFAM	$y=6.93x^{-0.108}$	−0.108
	CAM-A	$y=7.55x^{-0.110}$	−0.110
	HLAM-A	$y=7.64x^{-0.098}$	−0.098
	BFAM-A	$y=7.50x^{-0.111}$	−0.111
	HLBFAM-A	$y=8.43x^{-0.109}$	−0.109
650 με	CAM	$y=13.09x^{-0.137}$	−0.137
	HLAM	$y=12.93x^{-0.125}$	−0.125
	BFAM	$y=9.47x^{-0.086}$	−0.086
	HLBFAM	$y=12.21x^{-0.115}$	−0.115
	CAM-A	$y=11.87x^{-0.133}$	−0.133
	HLAM-A	$y=9.72x^{-0.101}$	−0.101
	BFAM-A	$y=10.76x^{-0.120}$	−0.120
	HLBFAM-A	$y=13.82x^{-0.130}$	−0.130

**Table 9:** Self-healing test results.

Specimen Types	Healing Conditions	Pre-Healing			Post-Healing		
		$N_{f1}\;$	$S_{1}\;(\mathrm{MPa})\;$	$S_{e1}\;(\mathrm{MPa})$	$N_{f2}\;$	$S_{2}\;(\mathrm{MPa})$	$S_{e2}\;(\mathrm{MPa})$
CAM	A	12,380	3946	1968	5071	3117	1543
	B	11,080	4129	2061	7719	3276	1599
	C	29,013	3516	1055	1581	2610	1303
HLAM	A	21,609	4993	2478	8634	3793	1862
	B	22,598	5219	2578	15,913	3913	1953
	C	54,908	5391	1617	2987	3792	1869
BFAM	A	18,213	3001	1497	6002	2381	1183
	B	15,435	3255	1601	9602	2481	1233
	C	42,193	3419	1026	4110	2019	1009
HLBFAM	A	20,011	4074	2009	7821	3276	1621
	B	19,374	4162	2079	14,821	3398	1701
	C	51,392	4510	1353	5992	2983	1490
CAM-A	A	6570	4501	2229	3080	4140	2053
	B	8314	4591	2302	4592	3917	1950
	C	18,920	4310	1293	774	2913	1456
HLAM-A	A	12,003	5173	2585	4910	4759	2374
	B	14,103	5617	2801	8913	4632	2302
	C	29,013	5201	1560	1102	3191	1590
BFAM-A	A	6819	3591	1795	3010	3329	1658
	B	7451	3699	1837	3910	3329	1649
	C	20,035	4013	1204	1620	2617	1353
HLBFAM-A	A	13,045	4310	2149	4310	4017	2004
	B	15,045	4263	2103	9310	3981	1974
	C	25,983	4983	1495	2410	3012	1503

## Data Availability

Data sharing is not applicable to this article.
